# Direct ultrastructural validation of endocrine cell identity in zebrafish pancreatic islets

**DOI:** 10.1007/s00441-026-04092-3

**Published:** 2026-09-05

**Authors:** N. Schmitner, C. Deseife, A. Neumann, W. Salvenmoser, A. C. Seybold, R. A. Kimmel, D. Meyer

**Affiliations:** 1 Institute of Molecular Biology, CMBI, University of Innsbruck, Innsbruck, Austria; 2 Institute of Zoology, CMBI, University of Innsbruck, Innsbruck, Austria

**Keywords:** Transmission electron microscopy, β-cells, Ablation, Endocrine pancreas, Zebrafish

## Abstract

The zebrafish is widely used to study glucose homeostasis, diabetes, and endocrine cell plasticity, yet a definitive ultrastructural characterization of adult pancreatic endocrine cell types has remained incomplete. Here, we combined transmission electron microscopy with hormone-specific immunogold labeling to define the ultrastructural characteristics of the major endocrine cell populations in adult zebrafish pancreatic islets. Three principal endocrine cell types were distinguished based on secretory granule morphology, electron density, and intracellular organization. Glucagon-positive α-cells contained electron-dense granules with a characteristic halo and eccentric dense core, insulin-positive β-cells displayed larger moderately electron-dense granules, and somatostatin-positive δ-cells possessed smaller, predominantly electron-dense granules of heterogeneous shape. Immunogold labeling for glucagon, insulin, and somatostatin linked granule ultrastructure to endocrine cell identity. To independently validate β-cell assignment, we performed nitroreductase-mediated β-cell ablation in juvenile zebrafish and observed a marked depletion of cells containing the characteristic medium electron-dense granules. Comparative analysis indicates that zebrafish endocrine granule ultrastructure differs in several respects from mammalian islets and exhibits similarities to selected teleost species, highlighting evolutionary diversity in endocrine granule organization. This study provides a foundational ultrastructural reference for the three major endocrine cell types of the adult zebrafish endocrine pancreas and establishes criteria for identifying these populations in studies of pancreatic disease, regeneration, and cell plasticity while also identifying an additional unassigned endocrine population that warrants further characterization.

**Supplementary Information:**

The online version contains supplementary material available at https://doi.org/10.1007/s00441-026-04092-3.

## Introduction

The pancreas is a vertebrate-specific endodermal organ executing major functions in food digestion and glucose homeostasis. The mature organ is composed of an exocrine compartment with acinar and duct cells that produce and transport digestive enzymes into the gut, and an endocrine compartment organized in pancreatic islets, from which metabolism-regulating peptide hormones are secreted into the blood stream. Pancreatic islets are compact clusters of endocrine cells embedded within the exocrine tissue, playing a critical role in the regulation of organism glucose homeostasis. Importantly, pancreas in mammalian systems and zebrafish have conserved physiological functions, reminiscent cellular compositions and structures, and conserved expression and function of most genes involved in pancreas development and maintenance (Argenton et al. 1999; Biemar et al. 2001; Eames et al. 2010; Jurczyk et al. 2011; Lin et al. 2004; Yee et al. 2005; Zecchin et al. 2004). The larval zebrafish pancreas is organized as an anterior head region containing the large, early developing principal islet, and a tail that expands posteriorly, containing later developing secondary islets. In the adult, the pancreatic structure shows increased complexity with multiple lobes of exocrine tissue extending along the intestine (Wan et al. 2006), within the exocrine tissue islets of highly variable size are embedded (Chen et al. 2007). The islet types include very large principal islets (> 150 µm in diameter), a structure called the Brockmann body, which is a collection of islets in close proximity, as well as diffusely distributed smaller secondary islets and single β-cells (Chen et al. 2007).

Although the zebrafish is extensively used to study glucose metabolism and pancreatic diseases, a complete ultrastructural analysis of the adult zebrafish pancreas by electron microscopy, identifying the main hormone producing endocrine cell types, is missing. Electron microscopy studies of zebrafish larvae have revealed endocrine cells containing electron-dense secretory granules within the pancreatic islet (Faraj et al. 2022). Three main types of secretory granules have been described in endocrine cells of the adult zebrafish pancreas (Chen et al. 2007). However, these ultrastructural observations were not combined with hormone-specific labeling to assign endocrine cell identity to specific granule morphologies. Comparisons with other teleost species indicate considerable variability in endocrine granule morphology (Table 1). In order to understand adult phenotypes in the islet that arise in the course of experimental manipulations and disease modelling, it is essential to have detailed knowledge of the wild type state.

**Table 1:** Comparative ultrastructural characteristics of endocrine secretory granules in teleost species

	α-cells	β-cells	δ-cells	Reference
Zebrafish	3 kinds of granules: 1. Round or oval-shaped, various size, moderate–strong electron density; 2. Surrounded by halo, elliptical or round, moderately electron dense; 3. Round or oval, bigger, less electron dense			Chen et al. (2007)
Sea bass	Variably sized, varying electron density, dense polygonal core, halo	Electron dense, occasionally rod-like core, no halo	Less numerous, smaller than insulin granules	Carrillo et al. (1986)
Sea bream	Round, 300 nm, polymorphic electron dense core, halo	Round, 410 nm, spherical electron dense core, halo	1. Round, 410 nm, medium electron density, no halo; 2. 290 nm, spherical electron dense core, halo	Abad et al. (1986)
Pufferfish	Round, 250–500 nm, hexagonal or tetragonal crystalline core 140–200 nm, high density, wide halo	Spherical, 240–400 nm, round compact core, moderate density, narrow halo	Spherical, 250–400 nm, low electron density, no halo	Kobayashi et al. (1976)
Catfish	Dense, spherical, of variable size, within smooth membraned sacs	Variable in size and shape (rectangular, square, hexagonal, irregular, round). in smooth-membraned sacs	Lower electron dense, larger in size	Luchini et al. (2015)

We hypothesized that the three previously described granule morphologies of the adult zebrafish endocrine pancreas (Chen et al. 2007) — granules with high electron density, medium electron density, and high electron density with a halo — correspond to distinct, hormonally defined α-cell, β-cell, and δ-cell populations. To test this, the ultrastructure of adult zebrafish pancreatic islets was analyzed by combining electron microscopy with immunogold labeling for confirmation of islet cell type. The specific aims of this study were as follows: (1) to characterize the ultrastructural features of the major secretory granule types in the adult zebrafish endocrine pancreas; (2) to assign hormone identity to each granule type using immunogold labeling for glucagon, insulin, and somatostatin; and (3) to independently confirm the β-cell assignment by targeted ablation of insulin-producing cells. Immunogold labeling for glucagon, insulin, and somatostatin enabled the correlation of the distinct granule morphologies with α-cell, β-cell, and δ-cell identities, and β-cell-specific ablation in 4-week-old zebrafish confirmed that cells with medium electron-dense granules were dramatically reduced upon loss of insulin-producing cells. Defining the ultrastructural histology of the three major pancreatic endocrine cells of zebrafish will provide an important reference for future studies to better define healthy and diseased states.

## Material and methods

### Zebrafish maintenance and fish lines

Zebrafish (*Danio rerio*) were maintained according to standard protocols. The fish lines used were wild-type Tuebingen, tg(ins:NTR-mcherry) (Li et al. 2017) expressing nitroreductase in insulin producing β-cells and tg(sst2:GFP) (Li et al. 2009) expressing the fluorophore eGFP in somatostatin producing cells.

### Hematoxylin and eosin histology

Adult fish (> 6 months) were euthanized, fixed in 4% paraformaldehyde, decalcified in BOUIN’s fluid for 10 days, embedded in paraplast wax and serially sectioned. Ten-micrometre-thick sections were stained with hematoxylin and eosin (H&E).

### Fixation and sample preparation for fluorescence microscopy

Adult (> 6 months) fish were anaesthetized for 5 min with Tricaine and euthanized on ice. Inner organs including the pancreas were dissected and fixated for 1 to 2 h in 4% PFA and embedded in 4% low melt agarose. Fifty-micrometre sections were cut with a Leica Vibratom and sections were collected for antibody staining. Sections were incubated in blocking buffer containing 1% DMSO, 1% sheep serum, 1% BSA, and 1% Triton X-100 in 1 × PBS for at least 60 min at room temperature. The sections were then incubated overnight at 4 °C with primary and secondary antibodies, at 1:200 and 1:1000 dilutions, respectively. Primary antibodies used were anti-insulin (guinea pig, A056401, Dako), anti-glucagon (mouse, G2654, Sigma), and anti-somatostatin (rabbit, A056601-2, Dako). Secondary antibodies are as follows: Alexa Fluor 405-conjugated anti-rabbit (detecting somatostatin), Alexa Fluor 546-conjugated anti-mouse (detecting glucagon), and Alexa Fluor 633-conjugated anti-guinea pig (detecting insulin) antibodies (Invitrogen; A31556, A-11030, A-21105). Confocal images were generated as previously described (Arkhipova et al. 2012) with a Zeiss LSM Exciter5 microscope equipped with 25 mW Argon laser (468, 488 and 514 nm), 1 mW 543 nm HeNe-laser, and a 5 mW 633 nm HeNe-laser.

### Fixation and sample preparation for electron microscopy

Adult fish were anaesthetized for 5 min with Tricaine and euthanized on ice. Inner organs including the pancreas, liver, and gut were dissected and fixed simultaneously according to Yee (Yee et al. 2005) using a mixture of paraformaldehyde and glutaraldehyde, followed by osmium tetroxide. Samples were dehydrated in an ascending acetone or alcohol series and embedded into EMBed 812 resin. Ultrathin sections were cut with a diamond knife (Diatome, Switzerland) using an ultra-microtome (Leica, Austria). Sections were stained with lead citrate and examined with a Zeiss Libra 120 energy filter transmission electron microscope (Zeiss, Germany). Images were acquired with a TRS 2 × 2 k high-speed camera (Tröndle, Germany) and ImageSP software (SYSPROG, Belarus).

### Fixation and sample preparation for immunogold staining

For immunohistochemistry, islets were dissected from tg(sst2:GFP) fish with delta cells labeled by GFP expression under fluorescent light. Tissue was fixated in 4% PFA overnight, dehydrated in an ethanol series, and gradually transferred into resin and embedded in LR-White. After polymerization, blocks were trimmed and sectioned as before. One hundred-nanometre-thick sections were then mounted on gold slot grids for immunogold processing. To detect antibody staining for insulin, glucagon, and somatostatin by TEM, we used a standard immunogold technique according to Hacker et al. (1996). Briefly, sections were treated with 5% BSA and 0.1% cold water fish gelatin for 60 min in 0.1 M PBS. Primary antibodies (guinea pig-anti-insulin (1:800, A056401, Dako), rabbit-anti-somatostatin (1:800, A056601-2, Dako), and rabbit-anti-glucagon (1:800, A0565, Dako)) were applied, each to a separate section, at room temperature for 2–3 h. After washing steps, a secondary gold-conjugated anti-rabbit (10 nm and 30 nm gold, 1:100 in FG/BSA; British Biocell, UK) or anti-guinea pig (10 nm, 1:100 in FG/BSA; British Biocell, UK) was applied for 1 h at room temperature. Glucagon-labeled sections were incubated with the 30 nm gold-conjugated anti-rabbit secondary, somatostatin-labeled sections with the 10 nm gold-conjugated anti-rabbit secondary, and insulin-labeled sections with the 10 nm gold-conjugated anti-guinea pig secondary. Sections were double stained with uranyl acetate and lead citrate and examined with a Zeiss Libra 120 electron microscope and images were taken with iTEM software (Olympus, Japan). For each staining procedure, negative controls were carried out by omitting the primary antibody and showed minimal background staining (Suppl. Figure 1).

### Β-cell ablation

Ablation of β cells in 4 wpf old zebrafish juveniles was performed by incubating the juveniles carrying the ins:NTR-mcherry transgene for 24 h with 7.5 µM Nifurpirinol (Bergemann et al. 2018). After inspection of ablation efficiency via fluorescence microscopy, whole juvenile fish were fixed according to Yee et al. (2005) and processed as previously described. Islet cellular composition was quantified on a single representative section per condition (control and β-cell-ablated). Cells of each type (α, β, δ, and unassigned) were manually outlined in Fiji (ImageJ) (Schindelin et al. 2012) based on granule morphology, and their combined area was expressed as a percentage of total islet area.

### Granule diameter measurement

Islet ultrastructure was examined in three adult zebrafish to confirm the consistency of granule morphology across individuals. Quantitative diameter measurement was performed on one representative animal to characterize the size range of each granule population. Granule diameters were subsequently measured manually in Fiji (ImageJ) using the straight line tool, calibrated to the scale bar of each electron micrograph, on several cells from several sections, yielding 27–30 measured granules per cell type. Because granule morphology (electron density and presence or absence of a halo) is itself the basis for cell-type classification, blinding to cell type during diameter measurement was not applicable; measurements were taken directly on identified granules within each classified cell type.

Quantitative diameter measurement of immunogold-labeled granules was performed on one representative animal to characterize the size range of each granule population. Granule diameters were subsequently measured manually in Fiji (ImageJ) using the straight line tool, calibrated to the scale bar of each electron micrograph, on several cells from two sections, yielding 32–35 measured granules per cell type.

Granule diameters are reported descriptively (mean ± SD) for each ultrastructurally and immunogold-confirmed cell type and were not subjected to statistical comparison between cell types, as cell-type identity was established independently through immunogold labeling and β-cell ablation rather than through granule size.

## Results

### Histology of the pancreas

**Fig. 1:**
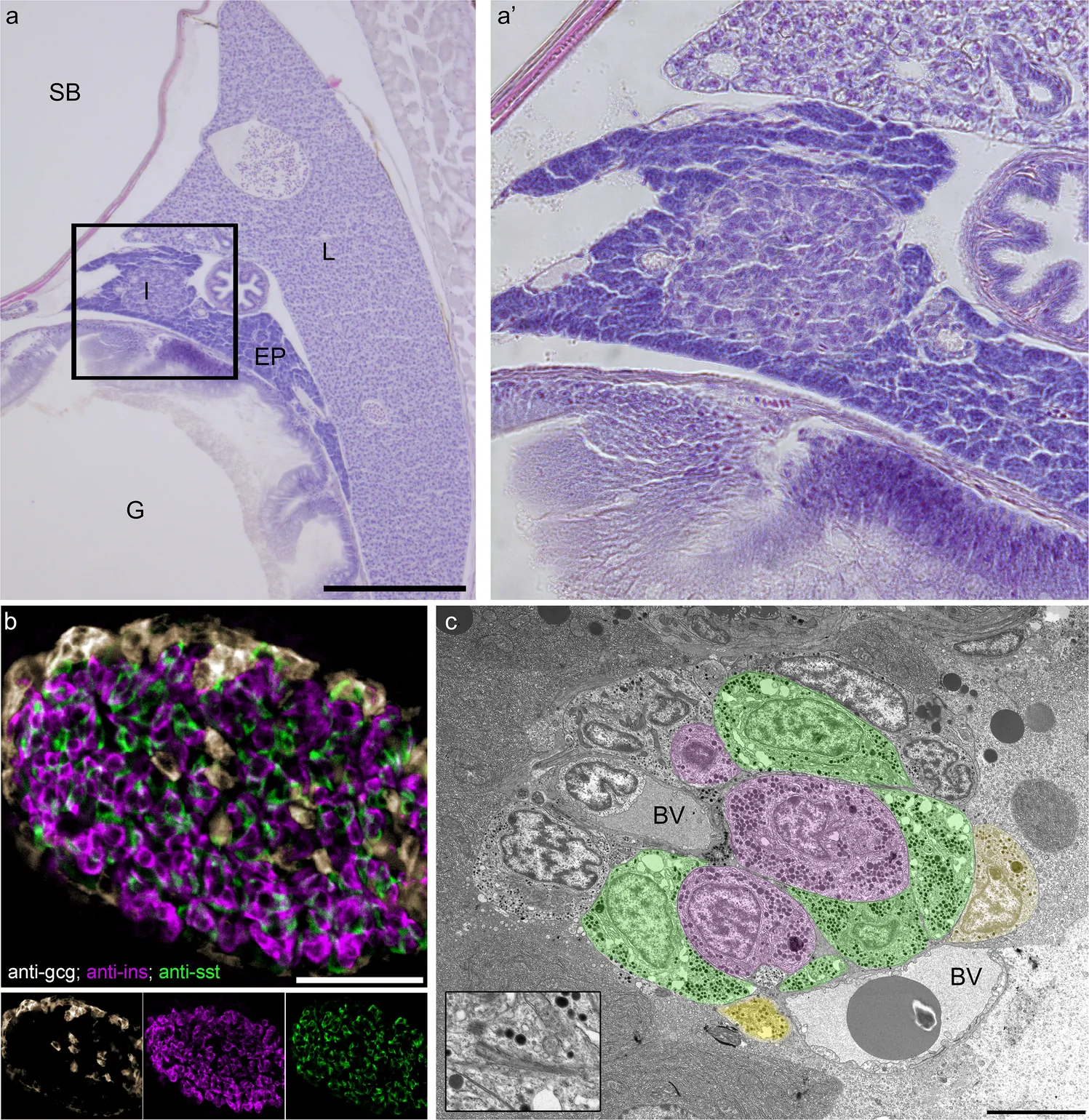
Organization of endocrine cell types in the zebrafish islet. **a** H&E stained transverse section through the abdominal region of an adult zebrafish showing the spatial organization of the pancreas relative to surrounding organs (L, liver; G, gut; SB, swim bladder; EP, exocrine pancreas; I, islet). Scale bar 200 µm. The boxed area denotes the region enlarged in panel **a’**. **a’** Boxed region in **a** highlighting the principal islet (I) embedded within pancreatic tissue. **b** Immunofluorescence staining showing the distribution of the major endocrine cell types in a primary islet of zebrafish. Shown are glucagon-positive α-cells (yellow), insulin-positive β-cells (magenta), and somatostatin-positive δ-cells (green). Scale bar 50 µm. **c** Transmission electron microscopy (TEM) overview of a secondary islet embedded within the exocrine pancreas. Cells with electron-dense granules with a halo (yellow), cells with large medium electron-dense granules (magenta), and cells with smaller electron-dense granules (green) are indicated, along with small capillaries (BV) and a red blood cell. High-magnification inset showing the primary cilium of a cell containing small, electron-dense granules, consistent with δ-cell identity as established in Section "Ultrastructural analysis of the endocrine pancreas". Scale bar 5 µm

In transverse sections, the adult zebrafish pancreas was located on the right body side, positioned between the gut and the liver (Fig. 1a). The endocrine part, consisting of one large Brockmann body or of two smaller principal islets and several secondary islets as well as isolated endocrine cells, was embedded in the more basophilic exocrine part of the pancreas. Immunofluorescence staining with hormone-specific antibodies revealed the main endocrine cell populations within the pancreatic islet, including glucagon-positive, insulin-positive, and somatostatin-positive cells (Fig. 1b). Glucagon positive α-cells were mainly distributed along the periphery, while insulin producing β-cells and somatostatin producing δ-cells were predominantly located in the central region of the islet.

### Ultrastructural analysis of the endocrine pancreas

**Fig. 2:**
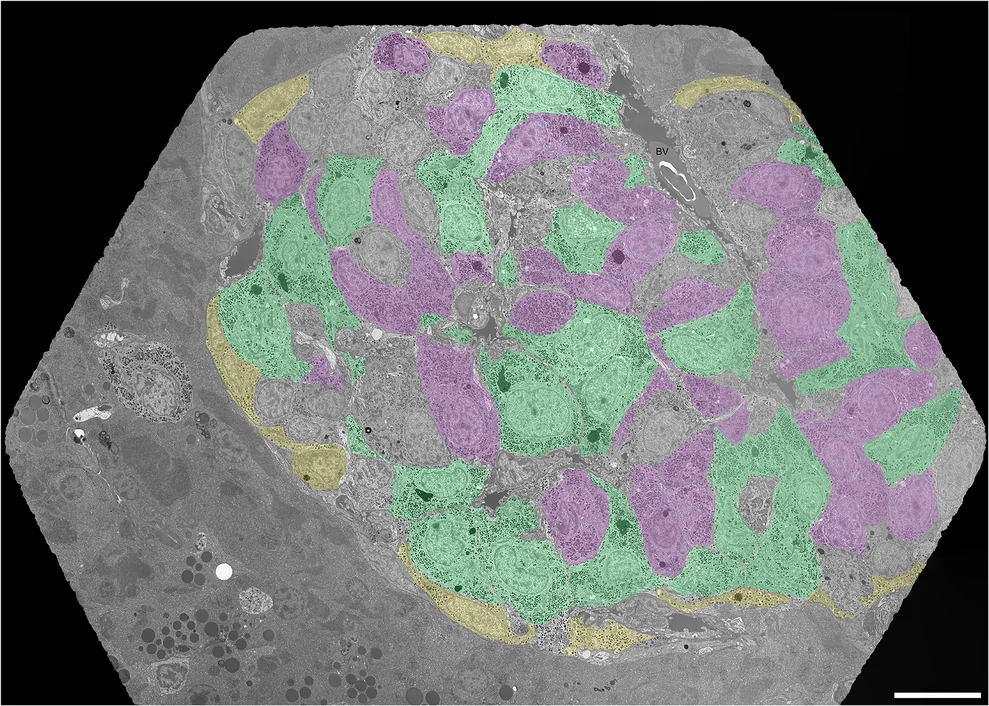
Ultrastructure of the principal zebrafish islet. TEM overview of a principal islet embedded in the exocrine pancreas. Cells with electron-dense granules with a halo (yellow), cells with large medium electron-dense granules (magenta), and cells with smaller electron-dense granules (green) are shown, together with small capillaries (BV) and a red blood cell. Scale bar 5 µm

Larger principal and smaller secondary islets were found embedded in exocrine tissue. The cells of the exocrine pancreas contained a prominent rough endoplasmic reticulum, large mitochondria, well developed Golgi-fields and huge secretory granules (Figs. 1c and 2). Capillaries associated with the islets showed fenestration of the endothelial cells. Within the islets, extracellular space was sparse and small nerve fibres with neuroactive vesicles were found occasionally. In the islet, we could differentiate several hormone-producing cell types based on their morphology, size of the granules, and their location within the islet (Figs. 1c and 2). At the cell surface of the hormone-producing cells, occasional cilium-like projections were observed (Fig. 1c inset).

**Fig. 3:**
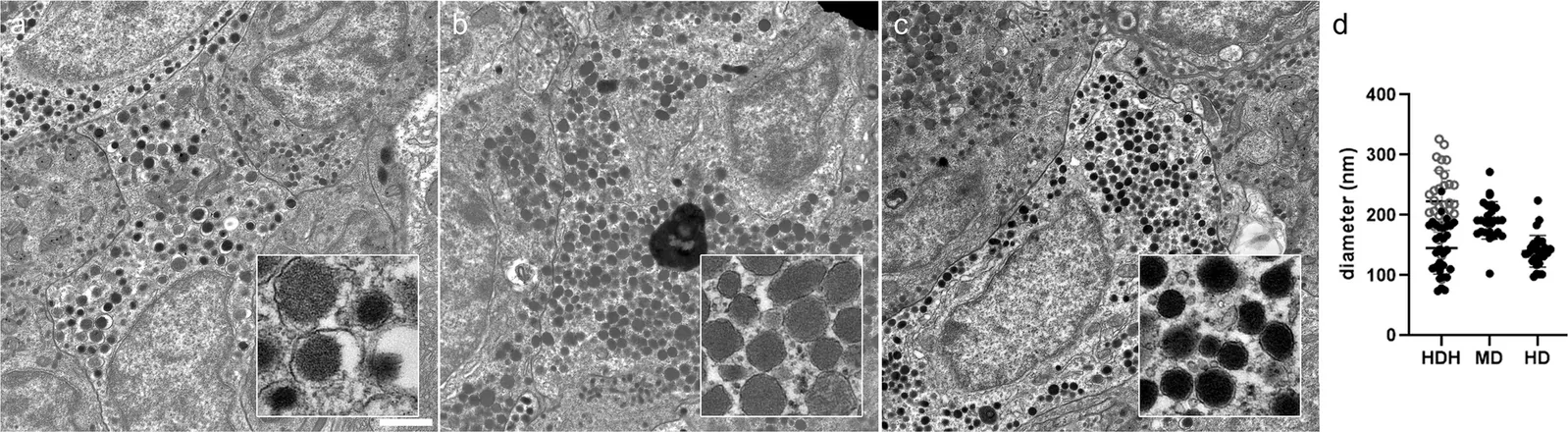
Ultrastructural characteristics of endocrine secretory granules. TEM images showing ultrastructural details of secretory granules in the major endocrine cell types of the zebrafish pancreas. **a** Cell containing characteristic granules with an electron-dense core and an electron-lucent halo. **b** Cell with large, moderately electron-dense granules. **c** Cell containing medium-sized electron-dense granules. Scale bar 1 µm. **d** Quantification of secretory granule diameters displayed as a dot plot with mean and SD indicated. Granules were categorized as high density with halo (HDH), medium density (MD), or high density (HD) based on electron density characteristics of the granule content. Granule diameters for cell type 1 (HDH), cell type 2 (MD), and cell type 3 (HD) were measured on one representative animal (islets examined qualitatively across *n* = 3 animals total); *n* = 30/27/30 granules per cell type, from several cells across several sections. Values are descriptive; not statistically compared between cell types

Cell type 1 was less numerous and mostly located at the periphery of the islet, appeared ellipsoid/oval or kidney shaped, and showed an eccentrically located nucleus, some mitochondria, and Golgi-fields, as well as some endoplasmic reticulum (Figs. 1c and 2, labeled in yellow). The secretory granules were spherical with an electron-dense core and a less dense mantle or a halo surrounded by a boundary membrane (Fig. 3a). These granules are hereafter referred to as high density with halo (HDH). The mean diameter of the granules was approximately 223 ± 52 nm (mean ± SD) and the core measured approximately 146 ± 43 nm (mean ± SD). Immature granules showed a less pronounced dense core and smaller bright halos.

Cell type 2, found at a high frequency distributed throughout the islet, appeared ellipsoid/oval with a centrally located nucleus. The nuclei were round to oval shaped and moderately euchromatic. The cytoplasm contained numerous oval to elongated mitochondria, prominent endoplasmic reticulum, well-developed Golgi fields, and in addition some lysosomes (Figs. 1c and 2, labeled in magenta). The cytoplasm appeared densely packed with secretory granules. The granules were spherical, medium electron-dense and showed a faint electron lucent mantle underneath the surrounding membrane and the mean diameter was 192 ± 31 nm (mean ± SD) (Fig. 3b). These granules are hereafter referred to as medium density (MD). Immature granules showed faint electron lucent substructures, sometimes also visible in mature granules.

Cell type 3 was also commonly found and distributed throughout the islet. They appeared ellipsoid/oval with a centrally located nucleus. The cytoplasm contained numerous oval to elongated mitochondria, Golgi fields, and most prominently a dilated, active endoplasmic reticulum (Figs. 1c and 2, labeled in green). The secretory granules appeared more heterogeneous in form and electron density. Generally, they were spherical and electron dense, some appeared oval or barbell shaped with a mean diameter of approx. 140 ± 26 nm (mean ± SD) (Fig. 3c). These granules are hereafter referred to as high density (HD). Some granules were larger and moderately electron dense.

Other cell types, either isolated or arranged in clusters, were found at the border and also in the centre of the islet. They had many smaller electron-dense granules or vesicles which appeared ellipsoid or spherical with a diameter ranging from 100 to 170 nm and showed a small electron lucent rim. These cells were not included in the granule diameter quantification shown in Fig. 3d and were not assigned a hormone identity, as no immunogold labeling was performed for additional candidate markers such as ghrelin, GIP (gastric inhibitory peptide), or PP (pancreatic polypeptide). We therefore cannot currently distinguish whether these cells comprise a rare endocrine subtype, have an immature or transitional cell state, or represent a non-endocrine cell type.

Granule morphology was examined across multiple adult zebrafish; quantitative granule diameter measurements (Fig. 3d) were subsequently performed on one representative animal and are reported here as descriptive characteristics of each granule population rather than as a formal statistical comparison between cell types (see Section "Granule diameter measurement" and Fig. 3).

### Immunogold labeling of pancreatic hormones

**Fig. 4:**
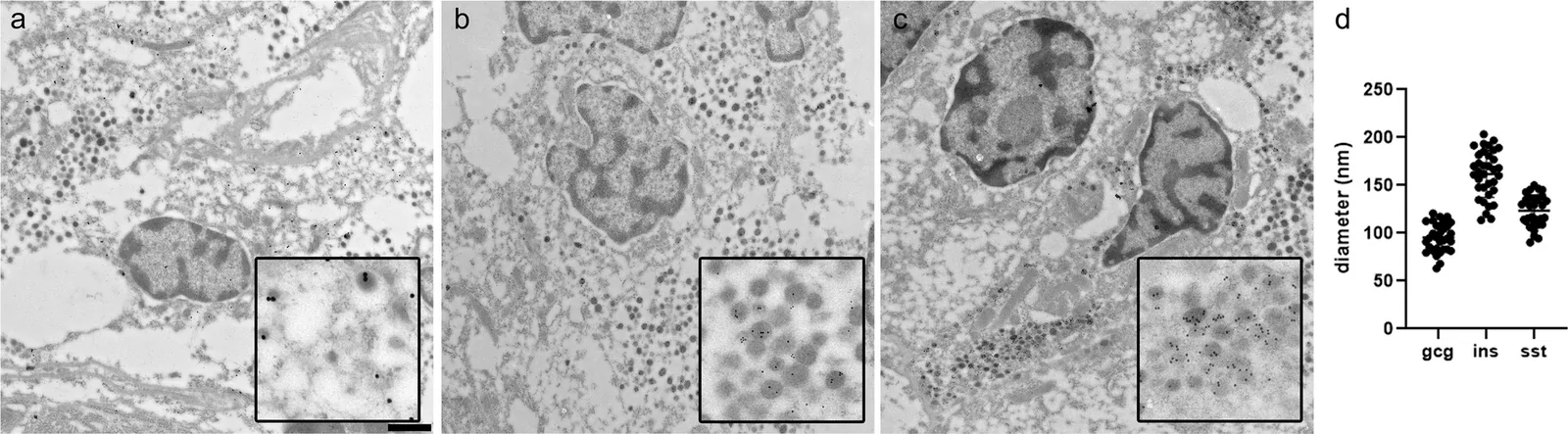
Immunogold labeling of islet hormones. Immunogold labeling of **a** glucagon, **b** insulin, and **c** somatostatin in endocrine cells of the zebrafish pancreas. Gold particles indicate specific hormone localization within secretory granules. Across all three hormones, only the dense granule core was labeled and measured; the surrounding halo/mantle and bounding membrane were not preserved due to omission of osmium fixation during immunogold preparation. Scale bar 1 µm. **d** Quantification of granule core diameters following immunogold labeling for glucagon, insulin, and somatostatin displayed as a dot plot with mean and SD indicated. Granule diameters were measured on one representative animal; *n* = 32/35/32 granules for glucagon/insulin/somatostatin, respectively, from several cells across two section per immunogold staining. Values are descriptive; not statistically compared between hormone types

Immunogold labeling was used to specify the three major hormones of the endocrine pancreas. For this, the fixation and embedding processes necessary for preserving antigen recognition altered granule contrast and morphology. Granule diameters were analyzed based on hormone-specific identity determined by immunogold labeling for glucagon (gcg), insulin (ins), and somatostatin (sst). It revealed that glucagon was located in the dense core of the granule with an average size of 95 ± 15 nm (mean ± SD) (Fig. 4a, d). Insulin was detected in the large granule cores of β-cells with an average size of 162 ± 25 nm (mean ± SD) (Fig. 4b, d). Somatostatin was seen in the electron-dense core of δ-cells with a granule size of 124 ± 16 nm (mean ± SD) (Fig. 4c, d). For all three hormones, only the granule core was measured, as the surrounding halo and bounding membrane were not preserved due to the absence of OsO₄ fixation required for immunogold antigen preservation. Immunogold labeling density, quantified as gold particles per granule area, was highest for somatostatin (332.3 particles/µm^2^, *n* = 32 granules) and insulin (266.5 particles/µm^2^, *n* = 35 granules), and lower for glucagon (126.1 particles/µm^2^, n = 32 granules), consistent with efficient and specific antibody labeling for all three hormones. Because the absolute granule dimensions measured by immunogold labeling are not directly comparable to those measured in conventionally fixed material, cell-type identity was established primarily by comparing the relative size ranking of granule populations within each preparation, rather than by matching absolute diameters across the two methods. Based on this relative ranking and location in the periphery of the islet, glucagon expressing α-cells were identified as cells containing electron-dense granules with a surrounding halo. Somatostatin-expressing δ-cells, which exhibited the smallest granules, were characterized by small, electron-dense granules with no obvious halo. Insulin-expressing β-cells were identified as cells containing medium electron-dense granules that were larger than somatostatin granules (summarized in Table 2).

**Table 2:** Correspondence between ultrastructurally defined cell types and hormone-confirmed endocrine cell identity in the adult zebrafish islet

Ultrastructural cell type	Hormone	Islet cell name	Granule morphology (conventional TEM)	Granule size (immunogold versus conventional TEM)	Structures lost in LR-White
Cell type 1	Glucagon	α-cell (glucagon-positive)	Electron-dense core with electron-lucent halo, bounding membrane (HDH)	~ 95 nm core (immunogold)/~ 146 nm core, ~ 223 nm total (conventional)	Halo, bounding membrane
Cell type 2	Insulin	β-cell (insulin-positive)	Medium electron-dense, faint lucent mantle beneath membrane (MD)	~ 162 nm (immunogold)/~ 192 nm (conventional)	Fine substructure/mantle contrast
Cell type 3	Somatostatin	δ-cell (somatostatin-positive)	Electron-dense, heterogeneous form, some oval/bar-bell shaped (HD)	~ 124 nm (immunogold)/~ 140 nm (conventional)	Fine granule contour detail
"Other cell types" (unassigned)	Not determined	Candidate ε-cell/GIP-cell/dlb + transitional population	Small electron-dense granules or vesicles, 100–170 nm, small electron-lucent rim	Not tested — no hormone-specific immunogold performed for this population	

### Ultrastructural analysis of the endocrine pancreas after β-cell ablation

**Fig. 5:**
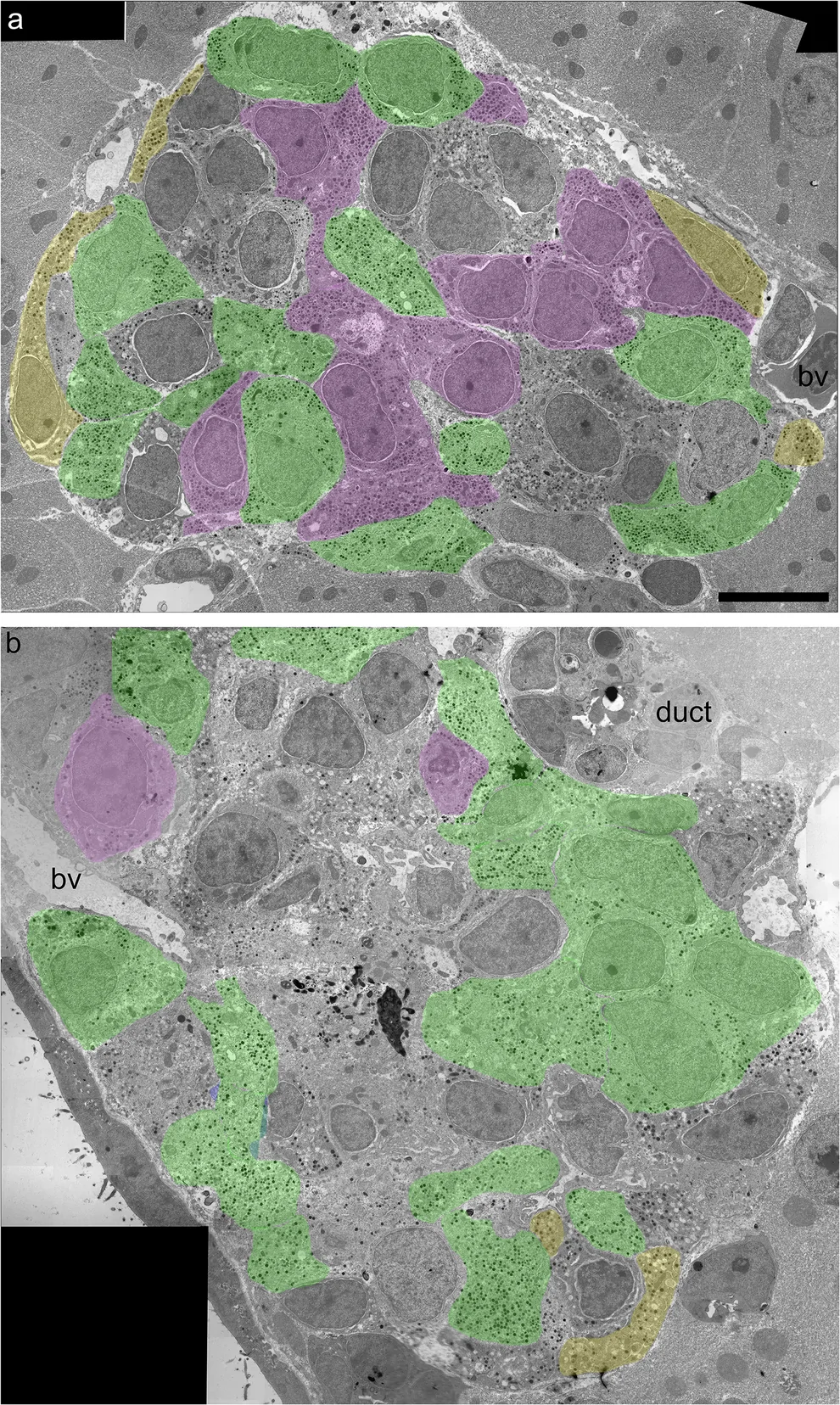
Structural comparison of control and β-cell–ablated islets. TEM images of the principal islet in a 4-week-old control fish (**a**) and an age-matched β-cell–ablated fish (**b**). Endocrine cells containing characteristic secretory granules are color-coded as follows: α cells (yellow), β cells (magenta), and δ cells (green). A pancreatic duct and small capillaries (BV) are indicated. The islet is embedded within the surrounding exocrine pancreas. Scale bar 5 µm

To confirm β-cell identity, ablation of β-cells was performed using tg(ins:NTR-mCherry) (Pisharath and Parsons 2009). Four-week-old transgenic zebrafish were either treated with DMSO or 7.5 µM Nifurpirinol. In control animals, the islet is composed of few α-cells (yellow) in the periphery of the islet, β-cells (magenta) and δ-cells (green), as well as some not further characterized cells in the core of the islet (Fig. 5a). The islet area not attributed to α-cells, β-cells, or δ-cells includes both this granule-containing unassigned population and other non-endocrine islet-associated tissue, such as ductal cells, immune cells, and vasculature, which were not separately quantified in the present analysis. Islets subjected to β-cell ablation showed a marked loss of β-cell mass (magenta). The islet architecture appeared disrupted compared to control, with a relative predominance of non-β endocrine cell populations. Quantification of islet cellular composition (below) is reported descriptively, as percentages reflect one representative islet per condition (control and β-cell-ablated). Based on their morphology and the presence of secretory granules, few residual β-cells were detectable (occupying 4% of total islet area compared to 26% in the control islet). These had only few secretory granules, which appeared smaller in size than the usual insulin granules. Occasional peripheral alpha cells (yellow) were present but remained sparse (2% in the ablated islet compared to 5% in the control islet). Delta cells, characterized by small electron-dense granules occupied 36% of total islet area compared to 31% in the control islet. The remaining islet area, comprising the unassigned granule-containing population together with non-endocrine tissue, accounted for approximately 38% of islet area in control and 58% in ablated islets, though these figures were not further broken down by cell type. The islet borders appeared less defined, and cellular density was reduced in regions where β-cells are absent. Overall, β-cell ablation resulted in a substantial depletion of cells with larger, medium electron-dense granules and therefore a corresponding shift in islet cellular composition and organization.

## Discussion

In both mammals and zebrafish, the fundamental composition and organization of the pancreatic islets are very similar. The pancreatic islet consists of a central part of insulin‐producing β-cells, surrounded by, in fish as in mammals, glucagon‐producing α‐cells, somatostatin-producing δ-cells, and ghrelin‐producing ε‐cells. Whether pancreatic polypeptide-producing PP‐cells also make up part of the zebrafish islet remains unclear, although PP cells have been described in a number of teleosts (Prince et al. 2017).

While fluorescence-based approaches allow analysis of endocrine cell identity and physiology in vivo, detailed ultrastructural characterization of endocrine granules in zebrafish islets remained limited (Faraj et al. 2022). In humans (Deconinck et al. 1971, 1972), other mammals (Lukinius et al. 1992; Shorr et al. 1970), and some teleost fish (Abad et al. 1986; Carrillo et al. 1986; Kobayashi et al. 1976; Luchini et al. 2015), endocrine cells have been distinguished through the distinct morphology of their secretory granules. In mammals, there is a general consensus about the granule types found in α-cells, β-cells, and δ-cells. In teleost fish like sea bass, sea bream, pufferfish, and catfish, granules found in the main cell types of the endocrine pancreas are more variably described concerning electron density, number, size, and possession of a halo (Table 1). Previously, three different cell types were identified by electron microscopy by their different kinds of secretory granules in zebrafish (Chen et al. 2007). One kind of granule in the islet cells was round or oval shaped, variable in size, and strongly or moderately electron dense; another kind of granule was surrounded by a halo, elliptical, or round and moderately electron dense; the third one was round or oval, bigger in size, and less electron dense. The characteristics of the granules did not match those found in humans and toads, and further identification of cells was not performed due to the lack of immune electron microscopy (Chen et al. 2007). Another reported ultrastructural observation of β-cell granules in zebrafish larvae showed moderately electron-dense granules (Yang et al. 2020), suggesting that insulin granule morphology differs from the classical halo-containing granules observed in mammals.

In this work, we characterized granule electron density based on conventionally fixed adult islets, and cell identity was assigned by immunogold labeling for insulin, glucagon, and somatostatin. Cell identity was independently supported by β-cell ablation, because antigen-preserving immunogold preparation does not retain comparable granule ultrastructure. The glucagon-containing granules of α-cells showed an eccentrically located dense core and a bright halo with a surrounding membrane in our adult zebrafish samples. This characteristic granule structure was also found in the sea bass, and immune electron microscopy revealed the location of glucagon on the dense core of the granule (Carrillo et al. 1986). Furthermore, the same structure of the α-cell granules was found in chicken (Mikami and Mutoh 1971), frog (Lozano et al. 1999), mouse and rat (Goping et al. 2003; Like et al. 1970; Mythili et al. 2004), and also in the human pancreatic islet (Deconinck et al. 1971, 1972).

Although mammalian β-cell granules typically display a highly electron-dense core with a peripheral halo, adult zebrafish β-cell granules appeared moderately electron dense under our fixation conditions. The ultrastructure of insulin-containing β-cells is reported to vary within teleost fish. In sea bass, the insulin-containing granules were also homogenous, but generally displayed an electron-dense core and no halo (Carrillo et al. 1986). In sea bream, the granules were spherical with a dense core and a halo (Abad et al. 1986), in pufferfish, they were round with a compact core of moderate electron density and a halo (Kobayashi et al. 1976), but in catfish, the granules showed crystalline inclusions (Luchini et al. 2015). Crystalline inclusions were quite frequent in β-cell granules of other vertebrate taxa and were reported from chicken, frog, mouse and rat, and also humans (Deconinck et al. 1971, 1972; Goping et al. 2003; Like et al. 1970; Lozano et al. 1999; Mikami and Mutoh 1971; Mythili et al. 2004; Pagliuca et al. 2014). Granules containing a dense core and a halo with a surrounding membrane could be generated by a chemical fixation artefact. High pressure freezing and freeze substitution of pancreas islets from rat revealed that the granules from β-cells lacked halos whereas chemically fixed β-cell granules appeared as hollow spheres with a halo (Fava et al. 2012).

Our classification of cell type 2 as β-cells was based on granule morphology and confirmed by insulin immunogold labeling and nitroreductase-mediated ablation, but does not exclude the presence of rare β/δ hybrid cells within this population. Single-cell transcriptomic studies of the zebrafish pancreas have identified insulin-positive cells with hybrid β/δ character, particularly during β-cell regeneration, although these represent only a minor fraction (~ 8%) of the insulin-positive population (Singh et al. 2022). Given this low prevalence, we consider it unlikely that hybrid cells substantially confound our granule morphology–based classification. Distinguishing such hybrid states ultrastructurally would require dual hormone immunogold labeling on the same section, which was beyond the scope of the present study, and is noted here as a limitation of the current classification.

The somatostatin-positive granules were consistently more electron dense and smaller in size compared to β-cell granules in adult zebrafish samples. The ultrastructure of somatostatin containing δ-cells is documented to be variable within teleost fish. In sea bass, granules were described as smaller than insulin granules (Carrillo et al. 1986), while in catfish, somatostatin granules were larger in size and less electron-dense (Luchini et al. 2015). In sea bream, two different δ-cell granules were observed, one being larger in size and medium electron-dense and the other smaller, with an electron-dense core and a halo (Abad et al. 1986). In chicken, less electron-dense granules surrounded by an indistinct membrane were reported; similarly in frog, the content of the granules was described as medium electron-dense (Lozano et al. 1999; Mikami and Mutoh 1971). In mouse and rat, δ-cell granules appeared variably electron dense and mostly missed a halo (Leiter et al. 1979; Goldsmith et al. 1975).

Several quantitative aspects of this study — granule diameters (Figs. 3d and 4d) and islet area fractions (Fig. 5) — were obtained from a single representative animal per condition and are reported descriptively rather than as statistically powered comparisons (Section "Granule diameter measurement").

The three ultrastructurally defined cell types identified here correspond to the major hormone-defined clusters reported in recent zebrafish pancreas single-cell transcriptomic atlases (Carril Pardo et al. 2022; Singh et al. 2022), which provides an independent, molecular line of support for our classification. These atlases also offer candidate molecular markers for the heterogeneity we observed within, and beyond, the three main cell types. Two transcriptomically distinct δ-cell populations, δ1 (*sst1.1⁺*) and δ2 (*sst2⁺*), differing in *pdx1* expression (Singh et al. 2022), could correspond to the heterogeneity in granule electron density and shape we observed within cell type 3 (Section "Ultrastructural analysis of the endocrine pancreas"), raising the possibility that our δ-cell population is ultrastructurally, as well as transcriptomically, heterogeneous. Notably, Singh et al. (2022) proposed, based on shared expression of the *pyyb*/Pyy paralogue and comparison with the evolutionary history of islet cell types (Heller 2010), that δ1-cells may represent the evolutionary counterpart of mammalian PP/γ-cells, raising the possibility that a PP-cell-like population is already represented within our δ-cell (cell type 3) classification rather than among the unassigned “other cell types”.

For the unassigned cell population described in Sections "Ultrastructural analysis of the endocrine pancreas" and "Ultrastructural analysis of the endocrine pancreas after β-cell ablation", several candidates emerge from these atlases. *ghrl⁺* ε-cells are an established zebrafish islet population not addressed by our immunogold panel. A further, well-supported candidate is a GIP-expressing population, which was identified as a transcriptionally distinct *gip⁺* cluster, separate from α-cells, β-cells, δ-cells, and ε-cells (Mi et al. 2023; Singh et al. 2022). Given that GIP is expressed in the zebrafish pancreas as well as the intestine, unlike in mammals where it is intestine restricted (Musson et al. 2009), this raises the possibility that the unassigned granules described here correspond, at least in part, to this GIP cell population. An additional candidate is the *dlb⁺* (*delta b* +) transitional endocrine precursor cells recently described by Mi et al. (2023). Testing these candidates directly would require immunogold or immunofluorescence labeling for the corresponding markers (Ghrelin, GIP, Dlb, Sst1.1 versus Sst2), combined with correlative ultrastructural imaging, which we propose as a direct next step for integrating the present granule-morphology-based reference with the transcriptomic landscape of the zebrafish islet.

Our study indicates species-specific differences in endocrine granule packaging, with the identity of hormones producing α-cells, β-cells, and δ-cells confirmed by immunogold labeling and β-cell ablation. Our ultrastructural identification and description of the main hormone-producing cells in the endocrine pancreas in the zebrafish will be useful for the characterization and analysis of the diseased pancreas in zebrafish. It can be used as a reference to further identify α-cells, β-cells, and δ-cells and to find evidence of de- and redifferentiation.

## Supplementary Information

Below is the link to the electronic supplementary material.

ESM 1 (DOCX 367 KB)

## Data Availability

All data generated or analyzed during this study are included in this published article. Additional data are available from the corresponding author upon reasonable request.
