# Supplementary material for: Direct ultrastructural validation of endocrine cell identity in zebrafish pancreatic islets
Source: Cell Tissue Res. 2026 Sep 5;405(2):8. doi: 10.1007/s00441-026-04092-3 (PMC13545134; doi:10.1007/s00441-026-04092-3)
Supplement: Supplementary file 1 [file 441_2026_4092_MOESM1_ESM.docx]

**Supplementary Fig. 1 Negative controls for immunogold labeling.** Representative TEM images of sections processed with the primary antibody omitted, using the same secondary gold-conjugated antibody as for the corresponding hormone: (A) anti-rabbit, 30 nm gold (glucagon negative control), (B) anti-guinea pig, 10 nm gold (insulin negative control), (C) anti-rabbit, 10 nm gold (somatostatin negative control). Background gold labelling was minimal (0, 13.2 and 18.4 particles/ µm² granule area for glucagon, insulin and somatostatin controls, respectively), confirming the specificity of the immunogold signal shown in Fig. 4. Scale bar: 500 nm.
